# Classifying Polyomavirus Nephropathy: The “Banff” Initiative

**DOI:** 10.3389/ti.2022.10299

**Published:** 2022-03-17

**Authors:** Volker Nickeleit, H. K. Singh, Vicki G. Davis, Surya V. Seshan

**Affiliations:** ^1^ Department of Pathology and Laboratory Medicine, Division of Nephropathology, The University of North Carolina School of Medicine, Chapel Hill, NC, United States; ^2^ Department of Pathology, Weill-Cornell Medical Center/New York Presbyterian Hospital, New York, NY, United States

**Keywords:** polyomavirus, PyVN, banff, validation, classification, biopsy, renal transplantation, outcome

Dear Editors,

All classification systems, especially if newly designed such as the Banff classification of polyomavirus nephropathy (PyVN) (1–3), have to be further validated. In this context, we read, with great interest, the article by Kowalewska et al. “*Assessment of the Banff Working Group classification of definitive BK polyomavirus nephropathy*” in the November 2021 issue of Transplant International (4). We are encouraged to learn about their findings confirming aspects of the Banff 3-tier polyomavirus nephropathy classification system. We are also not too surprised to read about some differences.

Based on statistical analysis, the Banff working group on polyomavirus nephropathy (here referred to as “Banff”) has identified two histologic variables, the ci and pvl scores as predictors of renal function (2, 3); those are used in the Banff system to define polyomavirus nephropathy disease classes (1). Kowalewska et al. reported similar observations (4). They also noted a significantly earlier diagnosis of PyVN in classes 1 and 2 compared to class 3. Post diagnosis all studies observed progressive deterioration of renal function in all PyVN classes, most pronounced in disease class 3. Both Banff studies (2, 3) and Kowalewska’s report (4) showed patients in disease class 3 with protracted viral resolution. Vice versa PyVN patients with disease resolution were more often found in disease classes 1 and 2. Interestingly, “Banff” (2) reported that early disease resolution indicated improved overall graft function and survival with most pronounced effects seen in class 2. Early clearance in class 2 (seen in 35% of cases) resulted in good outcome like class 1 and vice versa no clearance (in 65%) in inferior outcome like class 3 (2). Since Kowalewska et al. presumably were only able to collect a single serum creatinine data point post index biopsy at the 24-month mark, in contrast to the “Banff” reports with data collection at 1, 3, 6, 12, and 24 months, study results may not be fully comparable. However, there is general agreement among the studies that the detection of a lower PyVN class, often diagnosed early after transplantation, predicts good allograft function. In addition, early/efficient viral clearance and disease resolution are factors preserving graft integrity and stable S-Cr levels.

In order to assess the impact of a PyVN diagnosis on allograft function at time of the initial index biopsy, “Banff” compared the lowest S-Cr level before diagnosis (= best preceding baseline S-Cr) with the highest one at time of index biopsy/diagnosis, i.e. the maximum delta-change. Using this approach, “Banff” noted significant differences in function at time of diagnosis that were most pronounced in class 3. Kowalewka’s study design appears to have been less rigorous, presumably explaining the reported differences.

Differences between the studies were also seen in the graft failure rates that may most easily be explained by the applied definitions of “failure.” An additional aspect to consider in this context is the improved graft survival rate in PyVN observed over the last decade. In a PyVN patient cohort transplanted between 1996 and 2008 the overall graft failure rate within 24 months was 30% (3), compared to only 8% in a cohort transplanted post 2008 (2). A more favorable graft survival rate was also noted by Kowalewska et al. in their cohort of more recent kidney transplants with PyVN. Thus, in contrast to original studies presumably more reflective of the natural PyVN disease course (3), adaptations in patient management, such as regular screening of BK-DNAemia by PCR and early pre-emptive lowering of baseline immunosuppression (5, 6) have resulted in improved graft survival. Consequently, and not surprisingly the predictive power of PyVN disease classes to mark graft loss in current patient (2) compared to historic patient cohorts (3) is limited. “Not much failure can be predicted if loss is minor.” Very similar observations can be made with other disease entities, such as Banff type I rejection, where changes in patient management have resulted over time in improved clinical presentation and outcome.

We are surprised to learn about Kowalewska’s findings on BK-DNAemia in the PyVN classes. In both “Banff” studies different histologic viral load levels in class 1 (pvl-score: 1) versus class 3 (pvl-score: 3) resulted not surprisingly in significant differences in BK-DNAemia levels. Spearman’s rho, correlating histologic intra renal viral load levels, i.e. Banff pvl-scores, and BK-DNAemia is between 0.35 and 0.48 (7, 8). Thus, differences in BK-DNAemia between PyVN classes 1 and 3 are expected. Why Kowalewska et al. found very similar PCR reads in those PyVN classes in their study is undetermined; possibly differences in PCR test methodologies among centers are a reason (9, 10).

Any validation study faces challenges. Concurrent renal diseases, with rejection being one example, variations in inter observer lesion scoring, differences in PCR methodologies, or differences in study design can all influence data analysis and interpretation. We assume that Kowalewska et al., similar to “Banff,” exclusively used the time of the initial/first PyVN biopsy diagnosis as the primary reference point. We also assume that all cases of active and chronic rejection were excluded (although descriptions in their paragraph “characteristics of PyVAN classes” with “v,” “g,” and “cg” lesion scores render this assumption less clear). We also assume that the Banff ci-score/degree of interstitial fibrosis was evaluated in trichrome stains.

Kowalewska et al. conclude that PyVN “….classes do not correlate with the previously identified prognostic indicators such as interstitial inflammation or viral load.” Indeed, the “Banff” studies were not designed to confirm previous reports, but rather to propose a statistically based histologic PyVN classification system. By explicitly excluding cases of concurrent rejection and graft injury unrelated to PyVN, in depth statistical analyses did not reveal a significant association between interstitial inflammation and outcome. This “Banff” approach excluding confounding diseases differs significantly from other reports (11, 12). BK-DNAemia levels assessed by PCR allow for (diagnostic) risk stratification, i.e. low risk/high risk/presumptive PyVN. However, PCR test methodologies and results vary considerably, and the prognostic predictive value of BK-DNAemia levels is very limited (7, 8, 13). This notion was also confirmed in statistical analyses by “Banff” (see supplemental data (3)).

PyVN is a complication post kidney transplantation with major effects on allograft function. The “Banff” disease classes provide prognostic information. As Kowalewska et al. pointed out, their study approach mimicking “…the day-to-day practice” of pathology, is a very valuable contribution confirming some key findings of “Banff.” This day-to-day approach also illustrates that certain disease specific aspects are only uncovered in more rigorous studies. Thus, we interpret Kowalewka’s paper (4) as complementary to the Banff working group studies and report (1–3).
